# Disulfiram Protects Against Multiorgan Injuries and Cell Pyroptosis via Inhibiting GSDMD in Severe Acute Pancreatitis Mice

**DOI:** 10.1111/jcmm.70707

**Published:** 2025-08-13

**Authors:** Tianming Zhao, Si Zhao, Rui Fang, Yu Liu, Jing Ding, Xiaoxiao Shi, Shupei Li, Dan Xu, Xiaotan Dou, Mingdong Liu, Haijun Wan, Kang Jiang, Yuzheng Zhuge, Lei Wang, Hao Zhu, Lin Zhou

**Affiliations:** ^1^ Department of Gastroenterology Nanjing Drum Tower Hospital, Affiliated Hospital of Medical School, Nanjing University Nanjing Jiangsu China; ^2^ Department of Hyperbaric Oxygen Jinling Hospital, Affiliated Hospital of Medical School, Nanjing University Nanjing Jiangsu China; ^3^ Department of Gastroenterology and Hepatology Jinling Hospital, Affiliated Hospital of Medical School, Nanjing University Nanjing Jiangsu China

**Keywords:** disulfiram, gsdmd, multiple organ damage, NF‐ĸB, pyroptosis, severe acute pancreatitis

## Abstract

Severe acute pancreatitis (SAP) is distinguished by an uncontrolled systemic pro‐inflammatory response caused by the activation of trypsin within the pancreatic tissue, leading to the occurrence of multiple organ failure (MOF). Gasdermin D (GSDMD)‐induced pyroptosis represents a form of programmed cell death characterised by robust inflammatory responses. This indicates that directing efforts towards pyroptosis could potentially offer a remedy for SAP and its related MOF. Our objective was to examine the impact of disulfiram (DSF), a potent inhibitor of pyroptosis, and its potential therapeutic mechanism in SAP. The biochemical and histological assessments provided clear evidence that DSF effectively hindered necrosis, infiltration, oedema and cellular demise within pancreatic tissues. As a result, DSF effectively suppressed acute pancreatitis. Significantly, DSF hindered the process of GSDMD‐mediated pyroptosis in pancreatic cells within the context of SAP. This is evident through the observed decrease in the number of SYTOX‐positive cells, the prevention of LDH release and the restriction of expression of full‐length GSDMD, N‐terminal GSDMD and p‐NF‐ĸB p65. Subsequently, we assessed the mRNA levels of the pro‐inflammatory cytokines Il‐18, Il‐1β, Il‐6, Tnf‐α, Hmgb1 and Ccl2. Our findings revealed a significant rise in the levels of these pro‐inflammatory cytokines in SAP mice, whereas DSF remarkably inhibited the release of them. It is noteworthy that DSF also mitigated the resultant damage to remote vital organs (lungs, liver, and kidneys). Thus, GSDMD‐mediated pyroptosis has been significantly involved in the pathogenesis of SAP, and DSF could potentially serve as an alternative therapeutic agent for SAP and its associated MOF.

**Abbreviations**
ALT: alanine aminotransferase
AMY: amylase
BUN: blood urea nitrogen
Ccl2: chemokine (C‐C motif) ligand 2
DSF: Disulfiram
GSDMD: Gasdermin D
GSDMD‐FL: GSDMD full‐length
GSDMD‐N: cleaved gasdermin‐N domain
Hmgb1: high mobility group protein‐1
Il‐18: interleukin‐18
Il‐1β: interleukin‐1β
Il‐6: interleukin‐6
LDH: lactate dehydrogenase
LPS: lipopolysaccharide
MOF: multi‐organ failure
NF‐ĸB: nuclear factor‐kappaB
PBS: phosphate buffered saline
SAP: severe acute pancreatitis
TBIL: total bilirubin
Tnf‐α: tumour necrosis factor‐α

## 1 Introduction

Acute pancreatitis (AP) has the potential to cause pancreatic necrosis and inflammation, leading to subsequent multi‐organ failure (MOF) [1, 2, 3]. Severe acute pancreatitis (SAP) is correlated with elevated mortality rates and potentially life‐threatening complications [4, 5]. The severity of acute pancreatitis arises due to the transmigration and activation of leukocytes within the pancreas, along with the local synthesis and release of pro‐inflammatory‐soluble mediators. These factors lead to the conversion of a local injury into a systemic inflammatory response [6, 7]. According to the updated Atlanta classification system, injury frequently occurs in various distal major organs, such as the lungs, livers and kidneys, following the initiation of severe acute pancreatitis [8]. Nevertheless, the origin of SAP as well as its linked MOF continues to be unknown thus far.

Pyroptosis, which is a form of programmed necrosis, exhibits distinctive features such as the emergence of sizable vesicles from the plasma membrane, cellular breakdown and the discharge of pro‐inflammatory intracellular constituents [9]. Gasdermin family proteins are the direct cause of pyroptosis, as demonstrated by recent studies [10]. GSDMD, an element of the gasdermin family, serves as the substrate for caspase‐1 as well as caspase‐4/5/11 [11]. The cleavage of GSDMD into an N‐terminal GSDMD fragment (GSDMD‐N) occurs through the activation of caspase‐1 by various inflammasomes. The cleavage of GSDMD was similarly observed when caspase‐4/5/11 recognised cytosolic lipopolysaccharide (LPS), which constitutes the primary element of the gram‐negative bacterial cell wall [9]. GSDMD‐NT induces the formation of pores in the cell membrane, leading to cellular rupture and subsequent release of intracellular contents, such as IL‐18 and IL‐1β. This mechanism furthers the inflammatory response [10, 12]. The mechanism of pyroptotic cell death is constituted by the formation of GSDMD membrane pores. Multiple studies have demonstrated that pyroptosis may have a strong association with the occurrence of various diseases. Consequently, the inhibition of GSDMD emerges as an appealing approach to attenuating inflammation [13]. Recent advancements strongly indicate that targeting GSDMD‐mediated pyroptosis could potentially serve as an efficacious therapeutic approach for the treatment of AP. To illustrate, the initiation of pyroptosis and systemic inflammation in acute pancreatitis is facilitated through the activation of acinar cell NLRP3 inflammasome and GSDMD [14].

Disulfiram (DSF) is extensively utilised to address alcohol addiction [15, 16]. Disulfiram exhibited no adverse effects and was found to be well tolerated [17]. Disulfiram (DSF) could induce ferroptosis [18]. Recently, it has been reported that DSF has the ability to inhibit GSDMD‐induced pyroptosis [19]. This inhibition primarily occurs through the suppression of inflammatory caspase activation, GSDMD expression and GSDMD pore formation [13, 20, 21]. A successive investigation demonstrated that DSF had the capability to suppress the expressions of GSDMD and NETs within the living organism. Consequently, it alleviated SAP [22]. The severity of acute pancreatitis in mice is diminished by Disulfiram via inhibition of RIPK1‐dependent necrosis of acinar cells [23]. Furthermore, it was observed that disulfiram impeded the division of GSDMD, thereby mitigating caerulein‐induced severe acute pancreatitis (SAP) and associated pulmonary damage [24].

Nevertheless, there is a lack of information concerning the fundamental molecular mechanisms of DSF and its regulatory signalling within the SAP and its related MOF model. This study aims to investigate the potential protective impact of DSF in mice affected by SAP, while also elucidating the underlying mechanisms that contribute to this effect. In the present investigation, we created murine models of multiple organ failure associated with severe acute pancreatitis using caerulein in combination with lipopolysaccharide (LPS). These models demonstrated the activation of GSDMD‐mediated pyroptosis in various organs, namely the pancreas, lungs, liver, and kidneys. We have provided substantial evidence to support the significant effectiveness of disulfiram (DSF) in alleviating injuries sustained by multiple organs. Our discoveries indicate that pyroptosis, mediated by GSDMD, has a significant role in the development of SAP‐MOF. Additionally, DSF presents itself as a promising alternative therapeutic agent for SAP and its related MOF in order to impede cellular pyroptosis.

## 2 Materials and Methods

### 2.1 Chemicals and Reagents

Disulfiram was purchased from Rhawn, China (97‐77‐8). Caerulein was procured from GLPBIO, USA (GC30008). Lipopolysaccharide (LPS) was acquired from Beyotime, China (ST1470).

### 2.2 Animals

C57BL/6 wild‐type (WT) mice were acquired from Weitonglihua Biotechnology in Beijing, China. Briefly, male mice aged 8 weeks and weighing between 22 and 25 g were employed. The experiments were carried out in accordance with the guidelines stipulated by the Institutional Animal Committee of Nanjing Drum Tower Hospital.

### 2.3 Induction of Experimental Severe AP and Treatments

As presented in Figure S1, severe acute pancreatitis was induced with 8 hourly injections of caerulein (50 μg/kg, i.p.) and LPS (10 mg/kg, i.p. administered immediately after the last injection of caerulein) as previously described [25, 26, 27]. The PBS group was administered an equivalent quantity of phosphate buffered saline (PBS) injection. In the caerulein and LPS‐induced SAP model, mice were killed at 24 h after the last injection of LPS. Serum, pancreas, lungs, liver and kidneys were collected. DSF was conducted as previously described [28].

### 2.4 Statistical Analysis

The data were examined utilising either the two‐tailed Student’ *t*‐test or the one‐way ANOVA, followed by post hoc *t* tests. Unless specified differently, all data are presented as the mean ± standard deviation. A statistical significance threshold of *p* < 0.05 (*) was adopted for all tests. GraphPad Prism Version 9.0.0 was utilised to conduct the statistical analysis.

The Supporting Information contain information pertaining to additional materials and methodologies.

## 3 Results

### 3.1 Protective Effects of Disulfiram on Multiple Organ Injury in SAP Mice

**FIGURE 1:**
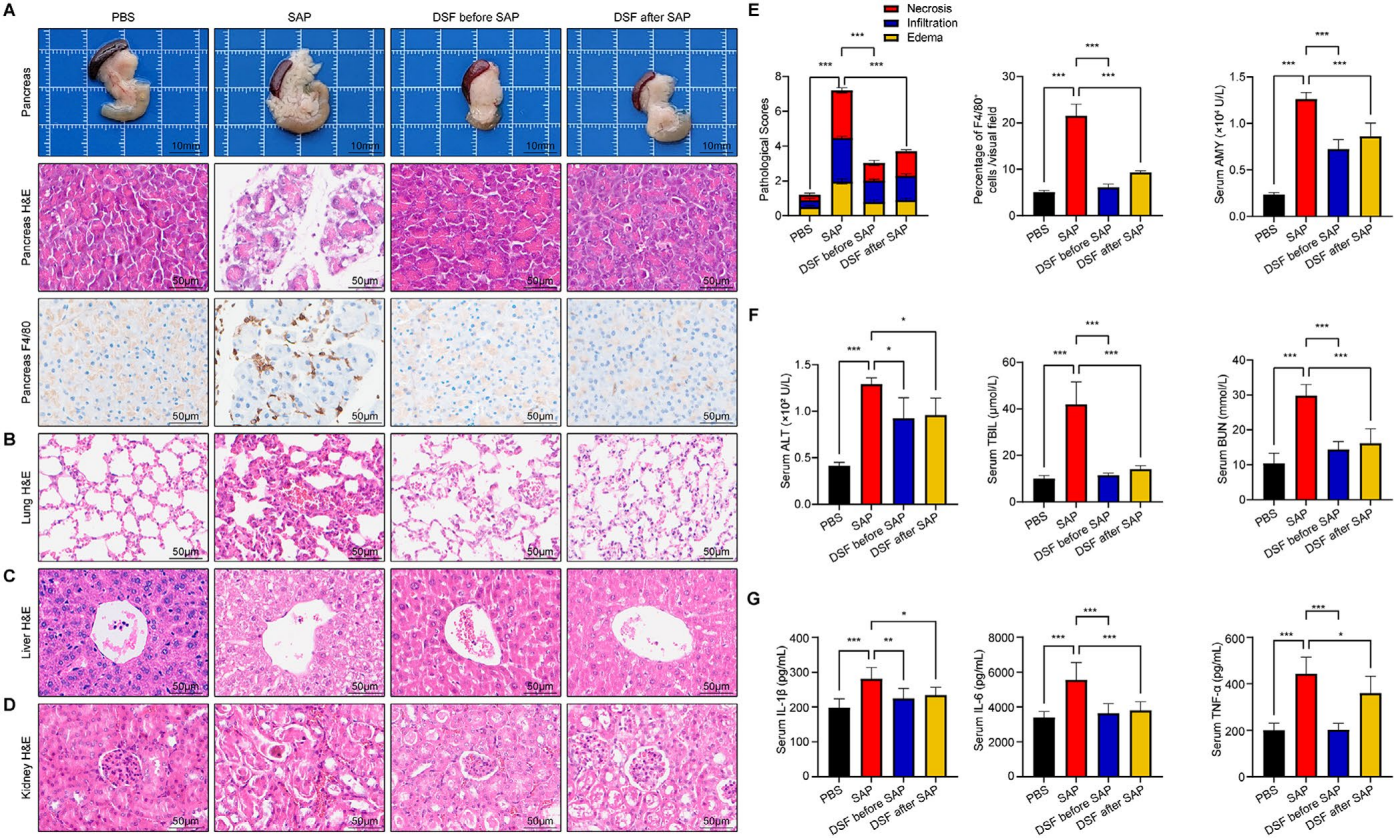
Organ damage in mice SAP model and multi‐organ therapeutic effects of disulfiram. (A) Gross morphology, H&E staining and F4/80 IHC staining of pancreas, (B) representative H&E stained lung sections, (C) representative H&E stained liver sections, (D) representative H&E stained kidney sections, (E) Histopathologic scores of the pancreas, quantitative data of F4/80^+^ cells of pancreas, serum AMY levels. (F) Serum ALT, TBIL and BUN levels. (G) Serum expression (ELISA) of IL‐1β, IL‐6 and TNF‐α. Data are expressed as mean ± SD, *n* = 6–8/group. *p < 0.05, **p < 0.01, ***p < 0.001.

The process of constructing our model has yielded positive results, as demonstrated in Figure 1. The mice SAP‐MOF model exhibits dysfunctions in the pancreas, lungs, liver and kidneys. The SAP group presents significantly increased interstitial tissue oedema, infiltration of inflammatory cells and necrosis or vacuolation in pancreatic acinar cells. Conversely, the PBS group showed no discernible pathological alterations. In contrast to the SAP group, both groups that underwent disulfiram treatment exhibited normal pancreatic tissue structure (Figure 1A). In order to assess macrophage infiltration within the pancreas, the tissues underwent staining for F4/80. A remarkable augmentation in the quantity of F4/80‐positive macrophages was observed in the pancreas affected by SAP. On the other hand, a pronounced decrease was observed in the infiltration of these cells in the groups undergoing disulfiram treatment (Figure 1E). In addition, the PBS group demonstrated a typical structure of the lungs. Nevertheless, the SAP group presented remarkable morphological changes, including the presence of alveolar oedema or collapse, as well as infiltration of inflammatory cells. On the contrary, both groups that received disulfiram treatment demonstrated a substantial enhancement in these elements (Figure 1B). Based on the findings of H&E staining, it was observed that the SAP group displayed infiltration of inflammatory cells and oedema in both the liver and kidney of mice. Nevertheless, such effects were alleviated in the presence of disulfiram (Figure 1C,D). The severity of pancreatitis was evaluated by measuring the pathology score. In the group with DSF before SAP, the pancreatic histological score was significantly lower compared to the SAP group (Figure 1E). Moreover, the score was also decreased in the DSF after SAP group. Moreover, a thorough examination was carried out on a range of enzymes. Compared to the control group administered with PBS, the levels of serum AMY, ALT, TBIL, BUN, IL‐1β, IL‐6 and TNF‐α exhibited an elevation in the group treated with SAP. It is worth mentioning that disulfiram significantly decreased the levels of AMY, ALT, TBIL, BUN, IL‐1β, IL‐6 and TNF‐α (Figure 1E–G). Based on the aforementioned findings, it can be deduced that disulfiram harbours the potential to alleviate multi‐organ damage induced by combining caerulein and LPS in mice.

### 3.2 Disulfiram Treatment Significantly Inhibites GSDMD‐Mediated Pancreatic Cell Pyroptosis

**FIGURE 2:**
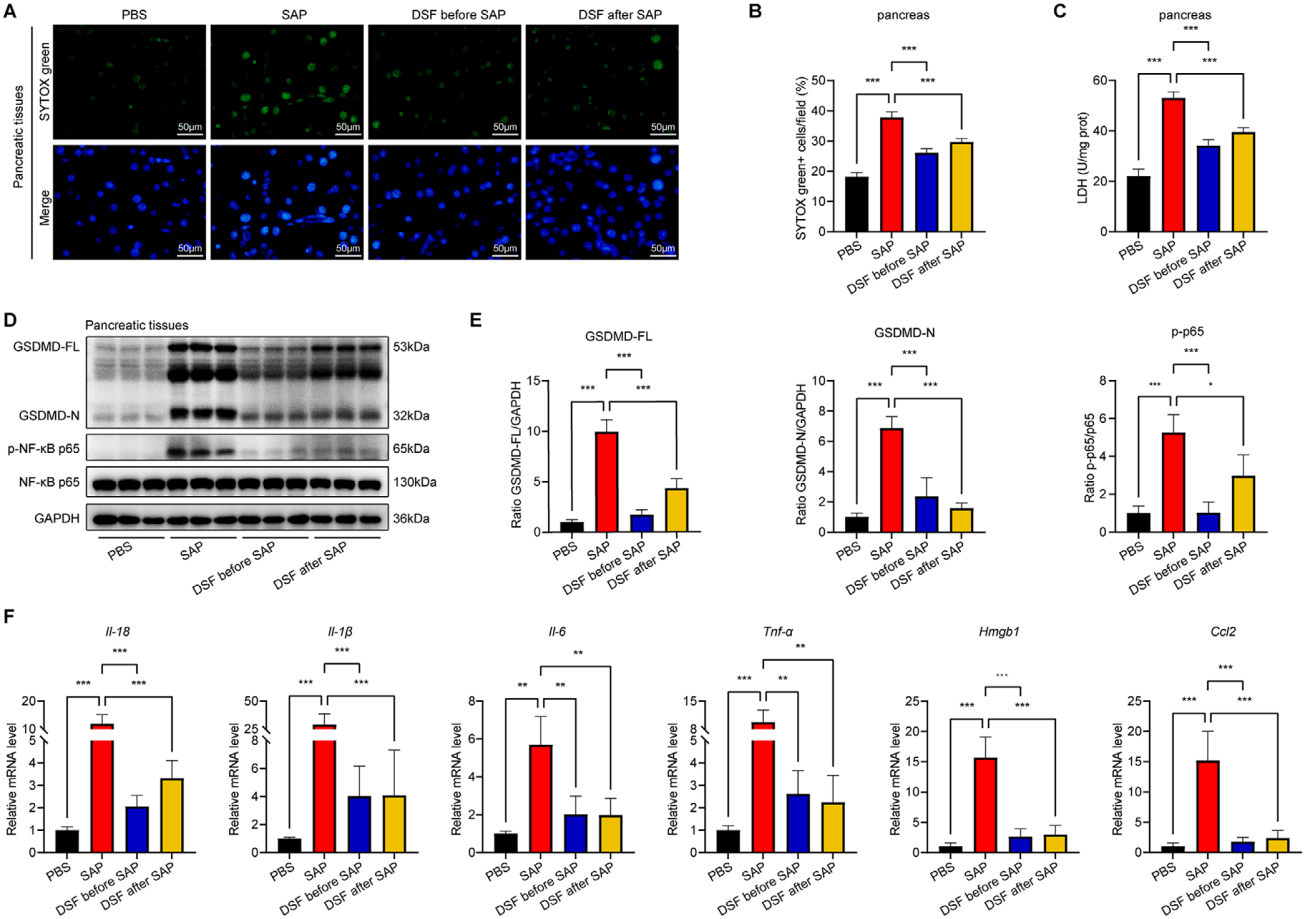
Effects of disulfiram on pancreatic damage in SAP mice. (A, B) Representative IF stained sections of SYTOX green in pancreas and quantitative data of SYTOX green^+^ cells, (C) LDH levels in pancreas tissues, (D, E) protein expressions of GSDMD, p‐NF‐κB and total‐NF‐κB in pancreas tissues, (F) pancreatic mRNA expression levels of *Il‐18, Il‐1β, Il‐6, Tnf‐α, Hmgb1* and *Ccl2*. Data are expressed as mean ± SD, *n* = 6–8/group. *p < 0.05, **p < 0.01, ***p < 0.001.

To gain a more comprehensive comprehension of the methodology through which disulfiram enhances the state of severe acute pancreatitis, we first conducted an analysis of cell death and LDH levels in the pancreatic tissues. The assessment of cellular demise was carried out by means of the absorption of SYTOX Green, whereby the number of cells exhibiting positive SYTOX Green staining was quantified. Surprisingly, the concurrent application of caerulein and LPS induced an increase in cellular mortality and LDH levels in the pancreas. However, the introduction of disulfiram showcased a significant suppression of cellular mortality and LDH levels (Figure 2A–C). There was a notable elevation in the protein levels of GSDMD‐FL and GSDMD‐N in SAP mice. Conversely, in the DSF before SAP group and DSF after SAP group, these protein levels exhibited a significant decrease. An appreciable increase in the protein level of p‐NF‐ĸB p65 was observed in mice with SAP. Consequently, the treatment groups that received disulfiram demonstrated a decrease in the expression of p‐NF‐ĸB p65 (Figure 2D and 2E). In order to further examine the function of GSDMD in SAP as an enhancer of pancreatic inflammation, we assessed the expression of pro‐inflammatory cytokines in the pancreas using real‐time quantitative polymerase chain reaction (RT‐qPCR). In the SAP group, the mRNA levels of *Il‐18*, *Il‐1β*, *Il‐6*, *Tnf‐α*, *Hmgb1* and *Ccl2* (*primers sequences used for qRT‐PCR are shown in* Table 1) exhibited a significant increase compared to the PBS group. It is worth noting that the application of disulfiram led to a suppression of the pro‐inflammatory cytokines (Figure 2F).

**TABLE 1:** List of primers for real‐time PCR.

Target	Gene ID	Primer	Sequence
*Il‐18*	16173	FP	5′‐GACAGCCTGTGTTCGAGGATATG −3′
		RP	5′‐TGTTCTTACAGGAGAGGGTAGAC‐3′
*Il‐1β*	16176	FP	5′‐TGGACCTTCCAGGATGAGGACA−3′
		RP	5′‐GTTCATCTCGGAGCCTGTAGTG−3′
*Il‐6*	16193	FP	5′‐TACCACTTCACAAGTCGGAGGC‐3′
		RP	5′‐CTGCAAGTGCATCATCGTTGTTC‐3′
*Tnf‐α*	21926	FP	5′‐GGTGCCTATGTCTCAGCCTCTT−3’
		RP	5′‐GCCATAGAACTGATGAGAGGGAG−3’
*Hmgb1*	15289	FP	5′‐CCAAGAAGTGCTCAGAGAGGTG−3’
		RP	5′‐GTCCTTGAACTTCTTTTTGGTCTC‐3’
*Ccl2*	20296	FP	5′‐GCTACAAGAGGATCACCAGCAG−3’
		RP	5′‐GTCTGGACCCATTCCTTCTTGG−3’
*Gapdh*	14433	FP	5′‐CATCACTGCCACCCAGAAGACTG−3’
		RP	5′‐ATGCCAGTGAGCTTCCCGTTCAG−3’

Abbreviations: FP, forward primer; RP, reverse primer.

### 3.3 Disulfiram Treatment Effectively Impedes Lung Cell Pyroptosis Induced by GSDMD

**FIGURE 3:**
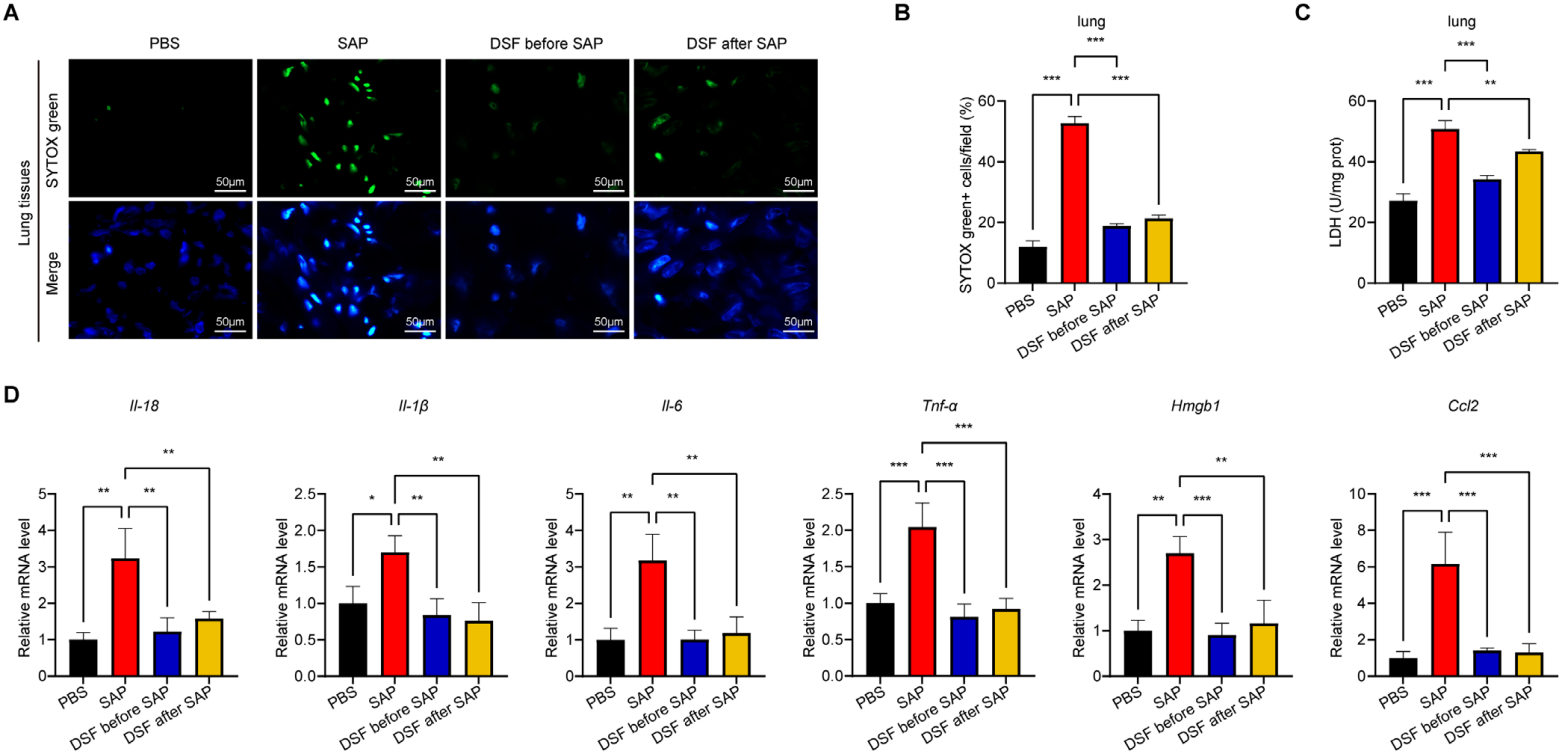
Effects of disulfiram on lung injury in SAP mice. (A and B) Representative IF stained sections of SYTOX green in lungs and quantitative data of SYTOX green^+^ cells; (C) LDH levels in lung tissues; (D) Pulmonary mRNA expression levels of *Il‐18, Il‐1β, Il‐6, Tnf‐α, Hmgb1* and *Ccl2*. Data are expressed as mean ± SD, *n* = 6–8/group. *p < 0.05, **p < 0.01, ***p < 0.001.

Afterward, we performed an analysis on the levels of cell death and lactate dehydrogenase (LDH) in the pulmonary tissues. Significantly, the SAP group displayed substantial cellular demise and increased levels of LDH. During our investigation, we discovered that the administration of disulfiram led to a notable decrease in lung cell mortality and LDH levels in the DSF before SAP group, in comparison to the SAP group. Furthermore, the DSF after SAP group exhibited commendable results (Figure 3A–C). Notably, the investigation of mRNA levels of *Il‐18*, *Il‐1β*, *Il‐6*, *Tnf‐α*, *Hmgb1* and *Ccl2* in the pulmonary tissues indicated a substantial increase in the SAP group compared to the PBS group (Figure 3D). Nevertheless, the administration of disulfiram effectively reinstated these levels to their typical condition.

### 3.4 Disulfiram Treatment Achieves Notable Inhibition of GSDMD‐Mediated Pyroptosis in Hepatocytes

**FIGURE 4:**
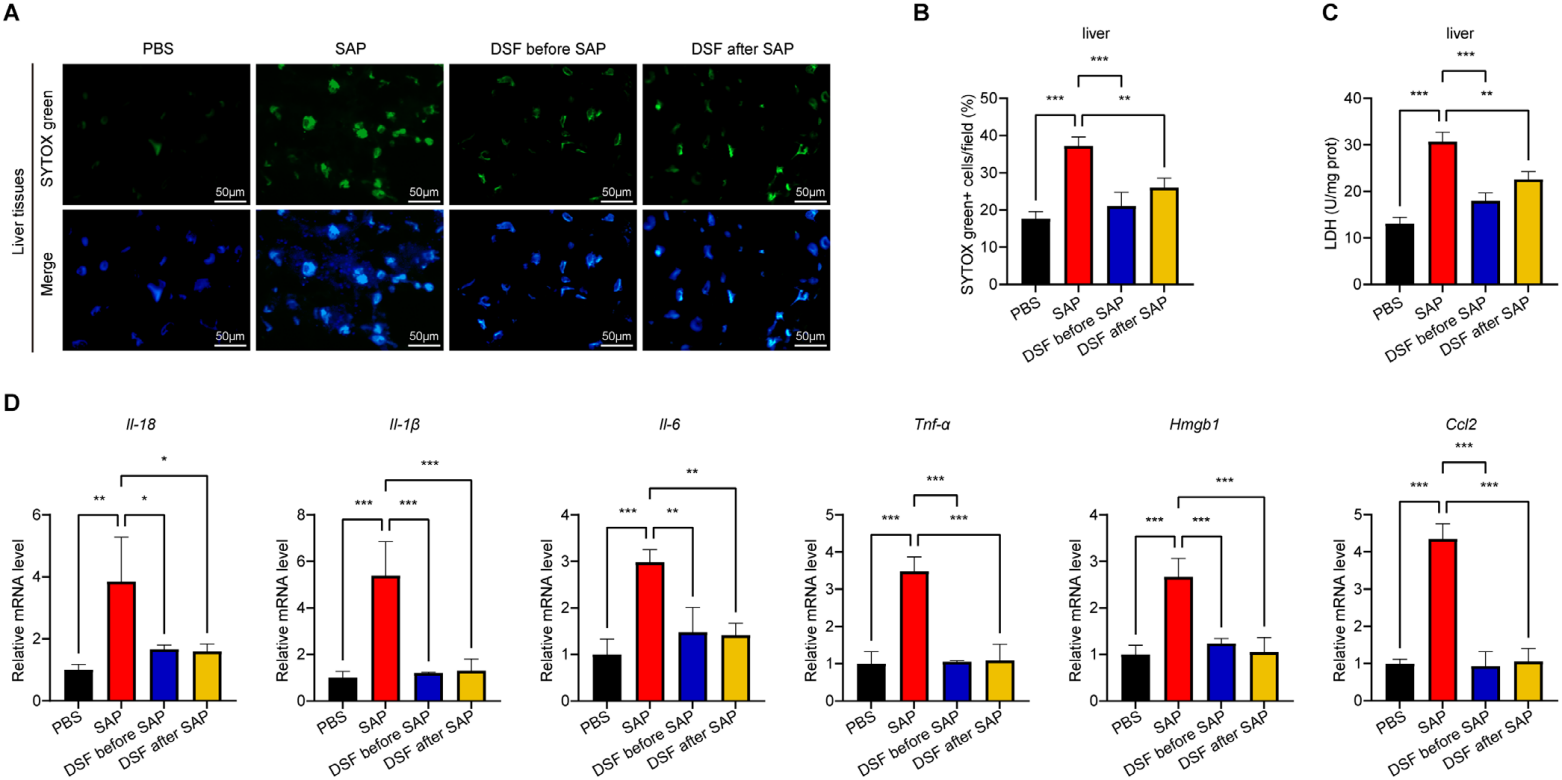
Effects of disulfiram on liver harm in SAP mice. (A and B) Representative IF stained sections of SYTOX green in livers and quantitive data of SYTOX green^+^ cells; (C) LDH levels in liver tissues; (D) Hepatic mRNA expression levels of *Il‐18, Il‐1β, Il‐6, Tnf‐α, Hmgb1*, and *Ccl2*. Data are expressed as mean ± SD, *n* = 6‐8/group. *p < 0.05, **p < 0.01, ***p < 0.001.

Following that, a comprehensive investigation was carried out on the cellular demise and levels of LDH in the hepatic tissues. It is worth mentioning that the SAP group displayed significant cellular demise and heightened LDH levels. As witnessed in our study, the administration of disulfiram led to a noteworthy decrease in lung cell demise and LDH levels compared to the SAP group (Figure 4A–C). Of particular significance, the mRNA levels of *Il‐18, Il‐1β, Il‐6, Tnf‐α, Hmgb1* and *Ccl2* in the hepatic tissues were significantly raised in the SAP group compared to the PBS group. However, the administration of disulfiram exhibited a noteworthy inhibition of this enhancement (Figure 4D).

### 3.5 Disulfiram Treatment Notably Hinders GSDMD‐Induced Pyroptosis in Renal Cells

**FIGURE 5:**
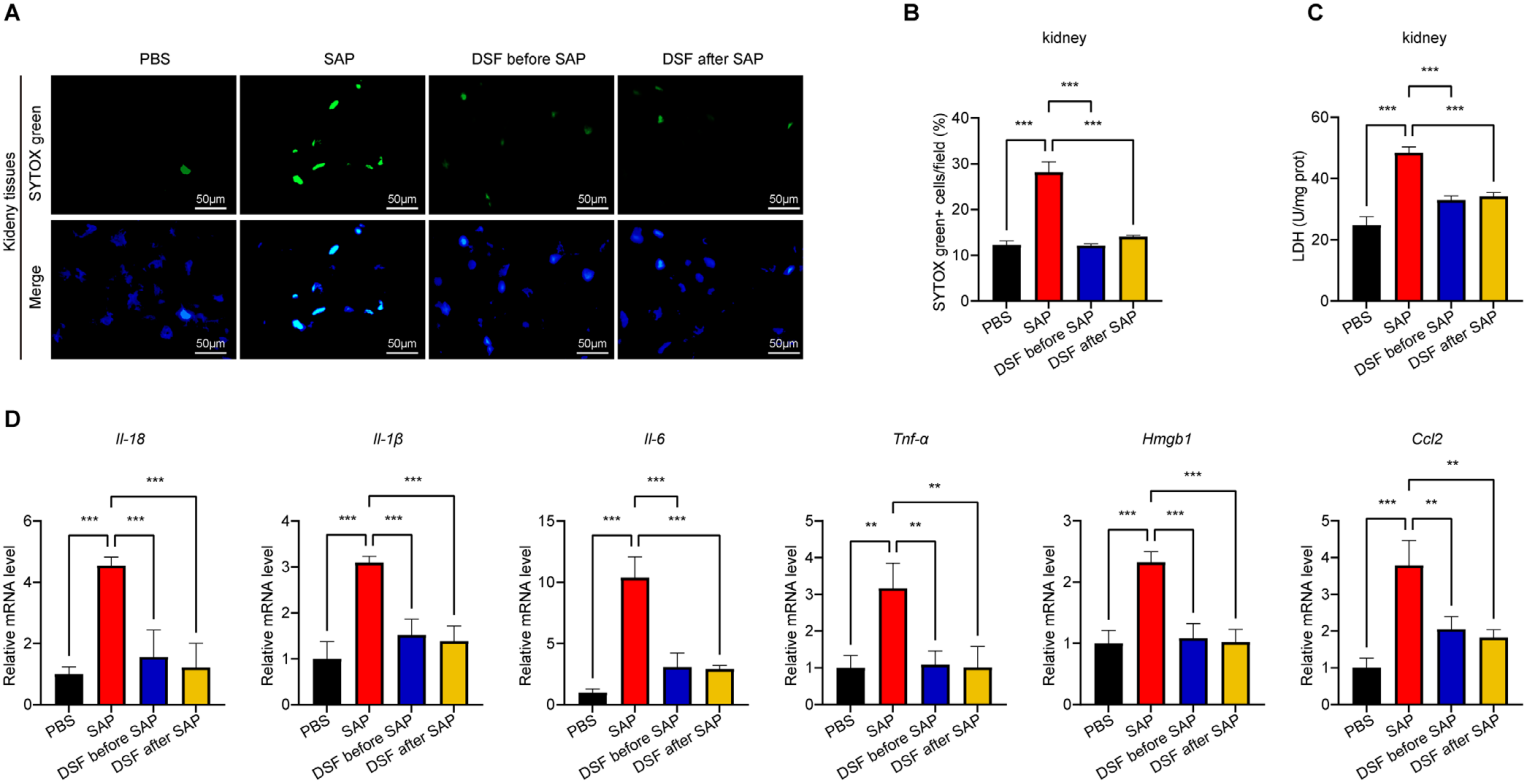
Effects of disulfiram on kidney impairment in SAP mice. (A and B) Representative IF stained sections of SYTOX green in kidneys and quantitative data of SYTOX green^+^ cells; (C) LDH levels in kidney tissues; (D) renal mRNA expression levels of *Il‐18, Il‐1β, Il‐6, Tnf‐α, Hmgb1* and *Ccl2*. Data are expressed as mean ± SD, *n* = 6–8/group. **p < 0.01, ***p < 0.001.

As a result, additional analysis was carried out regarding the incidence of cellular death and the levels of LDH in the kidney tissues. A substantial number of cellular death and increased LDH levels were observed in the SAP group. Intriguingly, the administration of disulfiram led to a significant decrease in renal cell death and LDH levels in comparison to the SAP group (Figure 5A–C). It is worth mentioning that the mRNA levels of *Il‐18, Il‐1β, Il‐6, Tnf‐α, Hmgb1* and *Ccl2* in the kidney tissues were significantly higher in the SAP group compared to the PBS group. Disulfiram administration also showed a notable reversal of this increment in kidneys (Figure 5D).

## 4 Discussion

In cases of acute pancreatitis, the presence of distal organ failure significantly increases the probability of mortality [29, 30]. The local inflammation of the pancreas possesses the capability to advance towards a systemic inflammatory response (SIRS), a common outcome being the manifestation of multiple organ dysfunction (MODS) and septic shock [31].

Disulfiram (DSF) has the potential to aid in the treatment of alcohol dependency by impeding the activity of aldehyde dehydrogenase (ALDH) [32]. Disulfiram possesses anti‐inflammatory properties that aid in the prevention of diverse types of cancers, including pancreatic cancer [33, 34]. Moreover, the drug disulfiram (DSF), which is used for treating alcoholism, exhibits strong inhibitory effects on pyroptosis. A study conducted in the past has presented compelling evidence suggesting that disulfiram effectively impedes pyroptosis and cytokine release in cells, as well as prevents septic death induced by lipopolysaccharide (LPS) in mice [13]. DSF has been recognised as an immensely effective protector for the pancreas. Disulfiram retards the generation of neutrophil extracellular traps (NETs) by suppressing gasdermin D (GSDMD), consequently mitigating the inflammation associated with SAP [22]. Disulfiram effectively suppressed the cleavage of GSDMD, ameliorated the development of caerulein‐induced severe acute pancreatitis (SAP) and the corresponding lung injury, and notably attenuated the expression levels of the pro‐inflammatory cytokines IL‐1β and IL‐18 [24]. The findings indicate that DSF, a highly effective inhibitor of pyroptosis, has the potential to be utilised as a suitable therapeutic strategy for managing severe acute pancreatitis.

However, there is a lack of information about the principal molecular apparatus of DSF and its regulatory signalling in the mice SAP‐MOF model. This study collectively intends to examine the potential safeguarding impact of disulfiram (DSF) on mice afflicted with severe acute pancreatitis, while also illuminating the underlying mechanisms. In this specific study, the researchers induced an experimental model of severe acute pancreatitis in mice by administering a combination of caerulein and LPS. Our objective was to investigate whether pyroptosis, mediated by GSDMD, played a role in the progression of SAP‐MOF. Furthermore, we aimed to ascertain the therapeutic efficacy of DSF in treating SAP and its associated MOF.

Caerulein is a cholecystokinin analogue that influences gallbladder contractions and induces the secretion of pancreatic enzymes [35, 36]. Additionally, it is responsible for the activation of trypsin from trypsinogen, subsequently triggering the autolysis of pancreatic acinar cells, which ultimately leads to the initiation of pancreatitis [37]. Lipopolysaccharide (LPS) is an endotoxin known for its capability to trigger a systemic inflammatory response by activating monocytes, subsequently resulting in the release of cytokines [38, 39, 40]. Bacterial lipopolysaccharide (LPS) serves as a powerful pathogen‐associated molecular pattern (PAMP) that initiates substantial inflammation in the context of sepsis [13]. The pyroptotic cell death that is facilitated by gasdermin D (GSDMD) pore formation is caused by the activation of the non‐canonic al inflammasome due to cytosolic lipopolysaccharide (LPS) [13]. Therefore, the simultaneous employment of caerulein and LPS demonstrates synergistic outcomes in the creation of a murine model that closely mimics SAP‐MOF.

In this present study, the SAP‐MOF model in mice shows dysfunctions in various organs, including the pancreas, lungs, liver and kidneys. DSF demonstrates the ability to mitigate the multi‐organ damage induced by caerulein and LPS in mice.

Disulfiram, alternatively recognised as antabuse, is an FDA‐approved medication for the treatment of alcohol dependency. It has been recognised as an inhibitor of GSDMD and pyroptosis [13, 41, 42, 43]. Additionally, it suppressed the in vitro and in vivo secretion of interleukin‐1β (IL‐1β) and interleukin‐18 (IL‐18) induced by lipopolysaccharide (LPS) [13]. Cell death pathways play a crucial role in host defence. Pyroptosis further exacerbates inflammatory conditions by facilitating the release of danger‐associated molecular patterns (DAMPs) [10]. The pore‐forming protein GSDMD is responsible for the execution of pyroptosis. The caspase cleavage of GSDMD results in the release of an N‐terminal p30 fragment, known as GSDMD‐N. This fragment subsequently undergoes oligomerisation and forms pores within the plasma membrane. The aforementioned pores function as a passage through which interleukin‐1β (IL‐1β) and IL‐18 are released, leading to the ultimate termination of the cell [10, 44]. NF‐κB is a transcriptional factor that regulates the expression of genes pivotal to diverse cellular processes, such as inflammation [45]. Prior research has further indicated that the phosphorylated NF‐κB p65 (p‐NF‐ĸB p65) subunit serves as a pivotal indicator of NF‐κB activation [45, 46]. The involvement of non‐canonical NF‐κB in pyroptosis is evident [47]. Previous studies have established that the activation of p‐NF‐κB p65 strongly correlates with IL‐1β and IL‐18 maturation, supporting the validity of our findings [43, 45]. Gasdermin D has the potential to modulate lipogenesis, the inflammatory response, and the NF‐κB signalling pathway [48].

To investigate the potential protective effect of disulfiram in mice affected by SAP and ascertain the involvement of the GSDMD pyroptosis pathway, an inquiry was conducted.

**FIGURE 6:**
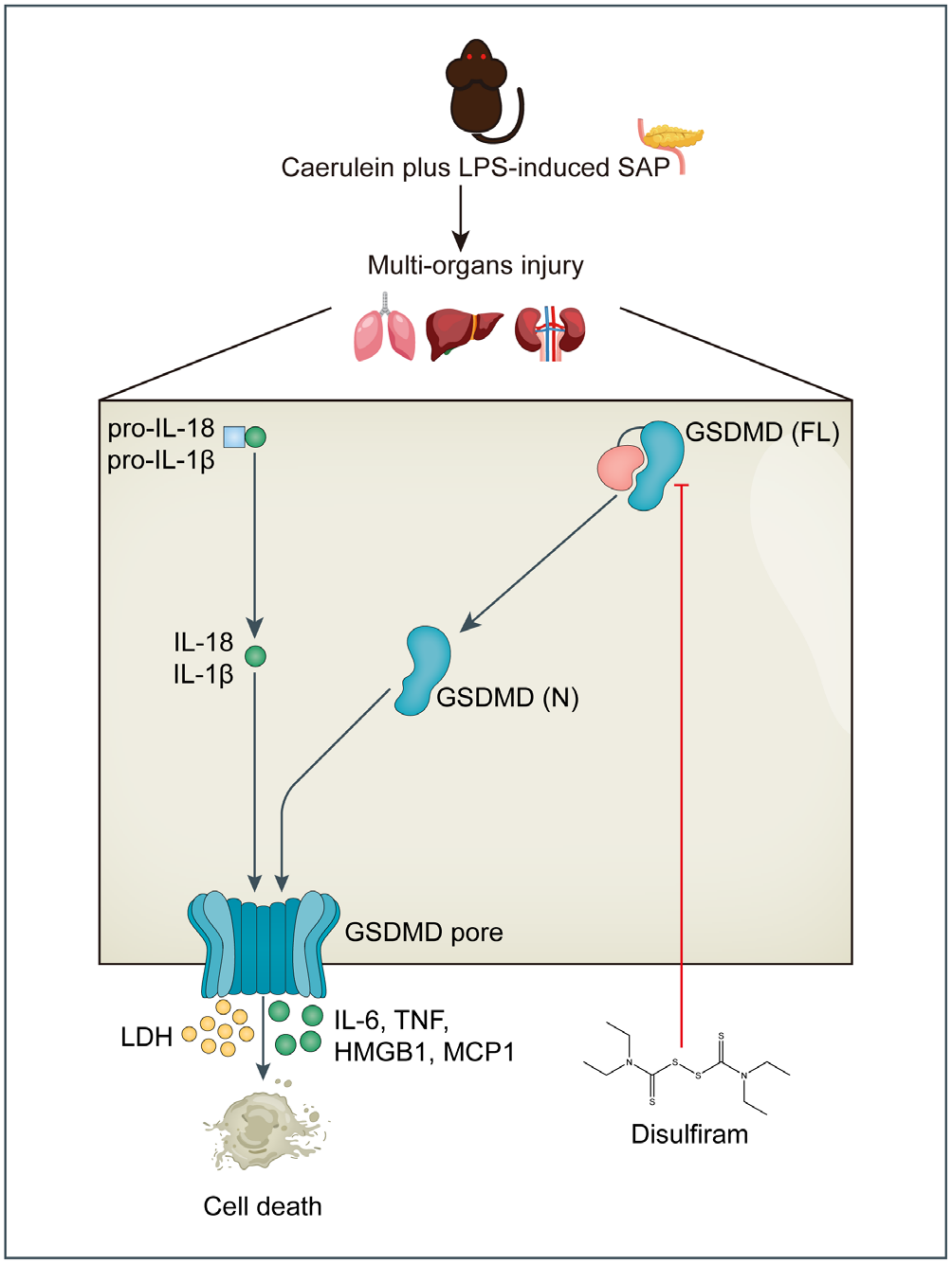
The diagram illustrates that disulfiram provides protection against multiorgan injuries and cell pyroptosis in severe acute pancreatitis mice by inhibiting GSDMD.

Our continuing research has uncovered the potential of disulfiram in treating multiple organ dysfunction associated with SAP by inhibiting the GSDMD pyroptosis pathway (Figure 6). Significantly, our findings demonstrate that disulfiram effectively inhibits GSDMD‐mediated pyroptosis of pancreatic cells in SAP. This is evident through a notable reduction in the number of SYTOX‐positive cells, prevention of LDH release, and the restricted expression of both GSDMD‐FL and GSDMD‐N. A notable rise in the protein level of p‐NF‐ĸB p65 was observed in mice with SAP. There was a significant increase in the protein level of p‐NF‐ĸB p65 in mice with SAP. Following this, the groups receiving disulfiram treatment demonstrated a decrease in the expression of p‐NF‐ĸB p65. Subsequently, we evaluated the mRNA expression levels of the pro‐inflammatory cytokines including *Il‐18, Il‐1β, Il‐6, Tnf‐α, Hmgb1* and *Ccl2*. In our study, it was discovered that there was an elevation in the levels of these pro‐inflammatory cytokines in mice with SAP. However, DSF exhibited a notable ability to suppress the release of these cytokines. Significantly, DSF also mitigated the resultant damage to prominent remote major organs, specifically the lungs, liver, and kidneys.

In caerulein‐induced mouse models, the absence of Gsdmd was found to improve the injury to the pancreas and related lungs caused by severe acute pancreatitis (SAP), as observed in the Gsdmd wild‐type SAP mouse models [24]. Prior research explored the role of DSF in severe acute pancreatitis and related lung injury. In contrast, our study delves deeper into the inhibition of pyroptosis, providing novel mechanistic insights into how DSF mitigates severe acute pancreatitis (SAP) and its associated multi‐organ failure [22]. Hence, GSDMD assumes a vital function in protecting cells from pyroptosis in the context of SAP.

## 5 Conclusion

To conclude, our findings indicate that GSDMD‐mediated pyroptosis is a viable therapeutic target in mice with severe acute pancreatitis (SAP), and disulfiram could potentially provide protective effects against SAP‐associated multiple organ failure (MOF). These discoveries offer novel insights into the molecular mechanisms underlying SAP and its related MOF as well as the potential for therapeutic interventions.

## Author Contributions

**Tianming Zhao:** conceptualization (equal), data curation (equal), formal analysis (equal), writing – original draft (equal). **Si Zhao:** conceptualization (equal), data curation (equal), formal analysis (equal). **Rui Fang:** conceptualization (equal), data curation (equal), formal analysis (equal). **Yu Liu:** data curation (equal). **Jing Ding:** conceptualization (equal), data curation (equal), formal analysis (equal). **Xiaoxiao Shi:** investigation (equal), methodology (equal). **Shupei Li:** investigation (equal), methodology (equal). **Dan Xu:** investigation (equal), methodology (equal). **Xiaotan Dou:** project administration (equal), resources (equal), software (equal). **Mingdong Liu:** project administration (equal), resources (equal), software (equal). **Haijun Wan:** supervision (equal), validation (equal), visualization (equal). **Kang Jiang:** supervision (equal), validation (equal), visualization (equal). **Yuzheng Zhuge:** supervision (equal). **Lei Wang:** supervision (equal). **Hao Zhu:** supervision (equal), validation (equal), visualization (equal). **Lin Zhou:** validation (equal), visualization (equal), writing – review and editing (equal).

## Ethics Statement

The authors have nothing to report.

## Conflicts of Interest

The authors declare no conflicts of interest.

## Supporting information


**FIGURE S1** Induction of experimental severe AP and treatments. (A) A schematic illustration of the severe acute pancreatitis model induced by caerulein plus LPS, **(B)** disulfiram was administrated before SAP, **(C)** disulfiram was administrated after SAP.

**Data S1.** Supporting Information.

## Data Availability

Data available on request from the authors.

## References

[1] D. R. J. Wolbrink , E. Kolwijck , J. ten Oever , K. D. Horvath , S. A. W. Bouwense , and J. A. Schouten , “Management of Infected Pancreatic Necrosis in the Intensive Care Unit: A Narrative Review,” Clinical Microbiology and Infection 26 (2020): 18–25, 10.1016/j.cmi.2019.06.017. 31238118

[2] A. Leppäniemi , M. Tolonen , A. Tarasconi , et al., “2019 WSES Guidelines for the Management of Severe Acute Pancreatitis,” World Journal of Emergency Surgery : WJES 14 (2019): 27, 10.1186/s13017-019-0247-0. 31210778 PMC6567462

[3] P. K. Garg and V. P. Singh , “Organ Failure due to Systemic Injury in Acute Pancreatitis,” Gastroenterology 156 (2019): 2008–2023, 10.1053/j.gastro.2018.12.041. 30768987 PMC6486861

[4] Y. S. Peng , C. S. Wu , Y. C. Chen , et al., “Critical Illness‐Related Corticosteroid Insufficiency in Patients With Severe Acute Biliary Pancreatitis: A Prospective Cohort Study,” Critical Care 13 (2009): R123, 10.1186/cc7978. 19630953 PMC2750175

[5] Y. Zheng , W. Sun , Z. Wang , et al., “Activation of Pancreatic Acinar FXR Protects Against Pancreatitis via Osgin1‐Mediated Restoration of Efficient Autophagy,” Research (Washington, D.C.) 2022 (2022): 9784081, 10.34133/2022/9784081. 36405253 PMC9667885

[6] J. P. Neoptolemos , M. Raraty , M. Finch , and R. Sutton , “Acute Pancreatitis: The Substantial Human and Financial Costs,” Gut 42 (1998): 886–891, 10.1136/gut.42.6.886. 9691932 PMC1727149

[7] L. Yamanel , M. Refik , B. Comert , et al., “The Effect of Activated Protein C on Experimental Acute Necrotizing Pancreatitis,” Critical Care 9 (2005): R184–R190, 10.1186/cc3485. 15987389 PMC1175873

[8] P. A. Banks , T. L. Bollen , C. Dervenis , et al., “Classification of Acute Pancreatitis‐2012: Revision of the Atlanta Classification and Definitions by International Consensus,” Gut 62 (2013): 102–111, 10.1136/gutjnl-2012-302779. 23100216

[9] J. Shi , W. Gao , and F. Shao , “Pyroptosis: Gasdermin‐Mediated Programmed Necrotic Cell Death,” Trends in Biochemical Sciences 42 (2017): 245–254, 10.1016/j.tibs.2016.10.004. 27932073

[10] J. Shi , Y. Zhao , K. Wang , et al., “Cleavage of GSDMD by Inflammatory Caspases Determines Pyroptotic Cell Death,” Nature 526 (2015): 660–665, 10.1038/nature15514. 26375003

[11] X. Li , P. Zhang , Z. Yin , et al., “Caspase‐1 and Gasdermin D Afford the Optimal Targets With Distinct Switching Strategies in NLRP1b Inflammasome‐Induced Cell Death,” Research (Washington, D.C.) (2022): 9838341, 10.34133/2022/9838341. 35958114 PMC9343085

[12] R. A. Aglietti and E. C. Dueber , “Recent Insights Into the Molecular Mechanisms Underlying Pyroptosis and Gasdermin Family Functions,” Trends in Immunology 38 (2017): 261–271, 10.1016/j.it.2017.01.003. 28196749

[13] J. J. Hu , X. Liu , S. Xia , et al., “FDA‐Approved Disulfiram Inhibits Pyroptosis by Blocking Gasdermin D Pore Formation,” Nature Immunology 21 (2020): 736–745, 10.1038/s41590-020-0669-6. 32367036 PMC7316630

[14] L. Gao , X. Dong , W. Gong , et al., “Acinar Cell NLRP3 Inflammasome and Gasdermin D (GSDMD) Activation Mediates Pyroptosis and Systemic Inflammation in Acute Pancreatitis,” British Journal of Pharmacology 178 (2021): 3533–3552, 10.1111/bph.15499. 33871879

[15] C. Wright and R. D. Moore , “Disulfiram Treatment of Alcoholism,” American Journal of Medicine 88 (1990): 647–655, 10.1016/0002-9343(90)90534-k. 2189310

[16] Y. Lei , L. Tang , Q. Chen , et al., “Disulfiram Ameliorates Nonalcoholic Steatohepatitis by Modulating the Gut Microbiota and Bile Acid Metabolism,” Nature Communications 13 (2022): 6862, 10.1038/s41467-022-34671-1. PMC9651870 36369291

[17] A. M. Spivak , A. Andrade , E. Eisele , et al., “A Pilot Study Assessing the Safety and Latency‐Reversing Activity of Disulfiram in HIV‐1‐Infected Adults on Antiretroviral Therapy,” Clinical Infectious Diseases 58 (2014): 883–890, 10.1093/cid/cit813. 24336828 PMC3935499

[18] C. Zeng , D. Nie , X. Wang , et al., “Combined Targeting of GPX4 and BCR‐ABL Tyrosine Kinase Selectively Compromises BCR‐ABL+ Leukemia Stem Cells,” Molecular Cancer 23 (2024): 240, 10.1186/s12943-024-02162-0. 39465372 PMC11514791

[19] J. Zhao , H. Wang , J. zhang , et al., “Disulfiram Alleviates Acute Lung Injury and Related Intestinal Mucosal Barrier Impairment by Targeting GSDMD‐Dependent Pyroptosis,” J Inflamm (Lond) 19 (2022): 17, 10.1186/s12950-022-00313-y. 36266722 PMC9582395

[20] J. Zhang , Y. Dai , Y. Yang , and J. Xu , “Calcitriol Alleviates Hyperosmotic Stress‐Induced Corneal Epithelial Cell Damage via Inhibiting the NLRP3‐ASC‐Caspase‐1‐GSDMD Pyroptosis Pathway in Dry Eye Disease,” Journal of Inflammation Research 14 (2021): 2955–2962, 10.2147/jir.S310116. 34262321 PMC8274828

[21] Y. Zhang , R. Zhang , and X. Han , “Disulfiram Inhibits Inflammation and Fibrosis in a Rat Unilateral Ureteral Obstruction Model by Inhibiting Gasdermin D Cleavage and Pyroptosis,” Inflammation Research 70 (2021): 543–552, 10.1007/s00011-021-01457-y. 33851234

[22] X. Ling , C. Nie , L. P. Sheng , C. Q. Han , and Z. Ding , “Disulfiram Relieves Severe Acute Pancreatitis by Inhibiting GSDMD‐Dependent NETs Formation,” Journal of Digestive Diseases 24 (2023): 359–368, 10.1111/1751-2980.13211. 37503822

[23] Q. Y. Huang , R. Zhang , Q. Y. Zhang , et al., “Disulfiram Reduces the Severity of Mouse Acute Pancreatitis by Inhibiting RIPK1‐Dependent Acinar Cell Necrosis,” Bioorganic Chemistry 133 (2023): 106382, 10.1016/j.bioorg.2023.106382. 36716580

[24] J. Wu , J. Zhang , J. Zhao , S. Chen , T. Zhou , and J. Xu , “Treatment of Severe Acute Pancreatitis and Related Lung Injury by Targeting Gasdermin D‐Mediated Pyroptosis,” Frontiers in Cell and Development Biology 9 (2021): 780142, 10.3389/fcell.2021.780142. PMC8632453 34858995

[25] Q. Zhang , J. Zhou , J. Zhou , R. H. Fang , W. Gao , and L. Zhang , “Lure‐and‐Kill Macrophage Nanoparticles Alleviate the Severity of Experimental Acute Pancreatitis,” Nature Communications 12 (2021): 4136, 10.1038/s41467-021-24447-4. PMC8260623 34230486

[26] H. Li , J. Xie , X. Guo , et al., “Bifidobacterium spp. and Their Metabolite Lactate Protect Against Acute Pancreatitis via Inhibition of Pancreatic and Systemic Inflammatory Responses,” Gut Microbes 14 (2022): 2127456, 10.1080/19490976.2022.2127456. 36195972 PMC9542615

[27] L. Kong , J. Deng , X. Zhou , et al., “Sitagliptin Activates the p62‐Keap1‐Nrf2 Signalling Pathway to Alleviate Oxidative Stress and Excessive Autophagy in Severe Acute Pancreatitis‐Related Acute Lung Injury,” Cell Death and Disease 12 (2021): 928, 10.1038/s41419-021-04227-0. 34635643 PMC8505515

[28] C. M. S. Silva , C. W. S. Wanderley , F. P. Veras , et al., “Gasdermin D Inhibition Prevents Multiple Organ Dysfunction During Sepsis by Blocking NET Formation,” Blood 138 (2021): 2702–2713, 10.1182/blood.2021011525. 34407544 PMC8703366

[29] P. Szatmary , T. Grammatikopoulos , W. Cai , et al., “Acute Pancreatitis: Diagnosis and Treatment,” Drugs 82 (2022): 1251–1276, 10.1007/s40265-022-01766-4. 36074322 PMC9454414

[30] M. S. Petrov , S. Shanbhag , M. Chakraborty , A. R. Phillips , and J. A. Windsor , “Organ Failure and Infection of Pancreatic Necrosis as Determinants of Mortality in Patients With Acute Pancreatitis,” Gastroenterology 139 (2010): 813–820, 10.1053/j.gastro.2010.06.010. 20540942

[31] A. S. Gukovskaya , I. Gukovsky , H. Algül , and A. Habtezion , “Autophagy, Inflammation, and Immune Dysfunction in the Pathogenesis of Pancreatitis,” Gastroenterology 153 (2017): 1212–1226, 10.1053/j.gastro.2017.08.071. 28918190 PMC6338477

[32] K. A. Hamblin , H. Flick‐Smith , K. B. Barnes , et al., “Disulfiram, an Alcohol Dependence Therapy, Can Inhibit the in Vitro Growth of *Francisella tularensis* ,” International Journal of Antimicrobial Agents 54 (2019): 85–88, 10.1016/j.ijantimicag.2019.04.002. 31029736

[33] S. Wadhwa and R. J. Mumper , “D‐Penicillamine and Other Low Molecular Weight Thiols: Review of Anticancer Effects and Related Mechanisms,” Cancer Letters 337 (2013): 8–21, 10.1016/j.canlet.2013.05.027. 23727371

[34] J. Cong , Y. Wang , X. Zhang , et al., “A Novel Chemoradiation Targeting Stem and Nonstem Pancreatic Cancer Cells by Repurposing Disulfiram,” Cancer Letters 409 (2017): 9–19, 10.1016/j.canlet.2017.08.028. 28864067

[35] V. P. Singh , A. K. Saluja , L. Bhagat , et al., “Phosphatidylinositol 3‐Kinase‐Dependent Activation of Trypsinogen Modulates the Severity of Acute Pancreatitis,” Journal of Clinical Investigation 108 (2001): 1387–1395, 10.1172/jci12874. 11696584 PMC209439

[36] A. K. Saluja , M. Saluja , H. Printz , A. Zavertnik , A. Sengupta , and M. L. Steer , “Experimental Pancreatitis Is Mediated by Low‐Affinity Cholecystokinin Receptors That Inhibit Digestive Enzyme Secretion,” Proceedings of the National Academy of Sciences of the United States of America 86 (1989): 8968–8971, 10.1073/pnas.86.22.8968. 2479032 PMC298412

[37] S. Wildi , J. Kleeff , J. Mayerle , et al., “Suppression of Transforming Growth Factor Beta Signalling Aborts Caerulein Induced Pancreatitis and Eliminates Restricted Stimulation at High Caerulein Concentrations,” Gut 56 (2007): 685–692, 10.1136/gut.2006.105833. 17135311 PMC1942167

[38] K. W. Moore , A. O'Garra , R. de Waal Malefyt , P. Vieira , and T. R. Mosmann , “Interleukin‐10,” Annual Review of Immunology 11 (1993): 165–190, 10.1146/annurev.iy.11.040193.001121. 8386517

[39] H. Qiu , A. S. Johansson , M. Sjöström , et al., “Differential Induction of BLT Receptor Expression on Human Endothelial Cells by Lipopolysaccharide, Cytokines, and Leukotriene B4,” Proceedings of the National Academy of Sciences of the United States of America 103 (2006): 6913–6918, 10.1073/pnas.0602208103. 16624877 PMC1440767

[40] Y. Hu , Y. Wang , X. Wen , et al., “Responsive Trimodal Probes for in Vivo Imaging of Liver Inflammation by Coassembly and GSH‐Driven Disassembly,” Research (Washington, D.C.) 2020 (2020): 4087069, 10.34133/2020/4087069. 33029587 PMC7520820

[41] S. Hu , L. Wang , Y. Xu , F. Li , and T. Wang , “Disulfiram Attenuates Hypoxia‐Induced Pulmonary Hypertension by Inhibiting GSDMD Cleavage and Pyroptosis in HPASMCs,” Respiratory Research 23 (2022): 353, 10.1186/s12931-022-02279-0. 36527086 PMC9756478

[42] S. Yang , Y. Feng , L. Chen , et al., “Disulfiram Accelerates Diabetic Foot Ulcer Healing by Blocking NET Formation via Suppressing the NLRP3/Caspase‐1/GSDMD Pathway,” Translational Research 254 (2023): 115–127, 10.1016/j.trsl.2022.10.008. 36336332

[43] C. Wang , T. Yang , J. Xiao , et al., “NLRP3 Inflammasome Activation Triggers Gasdermin D‐Independent Inflammation,” Science Immunology 6 (2021): eabj3859, 10.1126/sciimmunol.abj3859. 34678046 PMC8780201

[44] N. Kayagaki , I. B. Stowe , B. L. Lee , et al., “Caspase‐11 Cleaves Gasdermin D for Non‐Canonical Inflammasome Signalling,” Nature 526 (2015): 666–671, 10.1038/nature15541. 26375259

[45] T. Liu , L. Zhang , D. Joo , and S. C. Sun , “NF‐κB Signaling in Inflammation,” Signal Transduction and Targeted Therapy 2 (2017): 17023, 10.1038/sigtrans.2017.23. 29158945 PMC5661633

[46] P. C. Maity , T. Ray , B. Das , and A. K. Sil , “IKKβ‐I‐κBɛ‐c‐Rel/p50: A New Axis of NF‐κB Activation in Lung Epithelial Cells,” Oncogene 1 (2012): e8, 10.1038/oncsis.2012.8. PMC3412642 23552605

[47] X. Fan , Q. Li , Y. Wang , et al., “Non‐Canonical NF‐κB Contributes to Endothelial Pyroptosis and Atherogenesis Dependent on IRF‐1,” Translational Research 255 (2023): 1–13, 10.1016/j.trsl.2022.11.001. 36384204

[48] B. Xu , M. Jiang , Y. Chu , et al., “Gasdermin D Plays a Key Role as a Pyroptosis Executor of Non‐Alcoholic Steatohepatitis in Humans and Mice,” Journal of Hepatology 68 (2018): 773–782, 10.1016/j.jhep.2017.11.040. 29273476

