# Supplementary figures and images for: Disulfiram Protects Against Multiorgan Injuries and Cell Pyroptosis via Inhibiting GSDMD in Severe Acute Pancreatitis Mice
Source: J Cell Mol Med. 2025 Aug 13;29(15):e70707. doi: 10.1111/jcmm.70707 (PMC12344577; doi:10.1111/jcmm.70707)

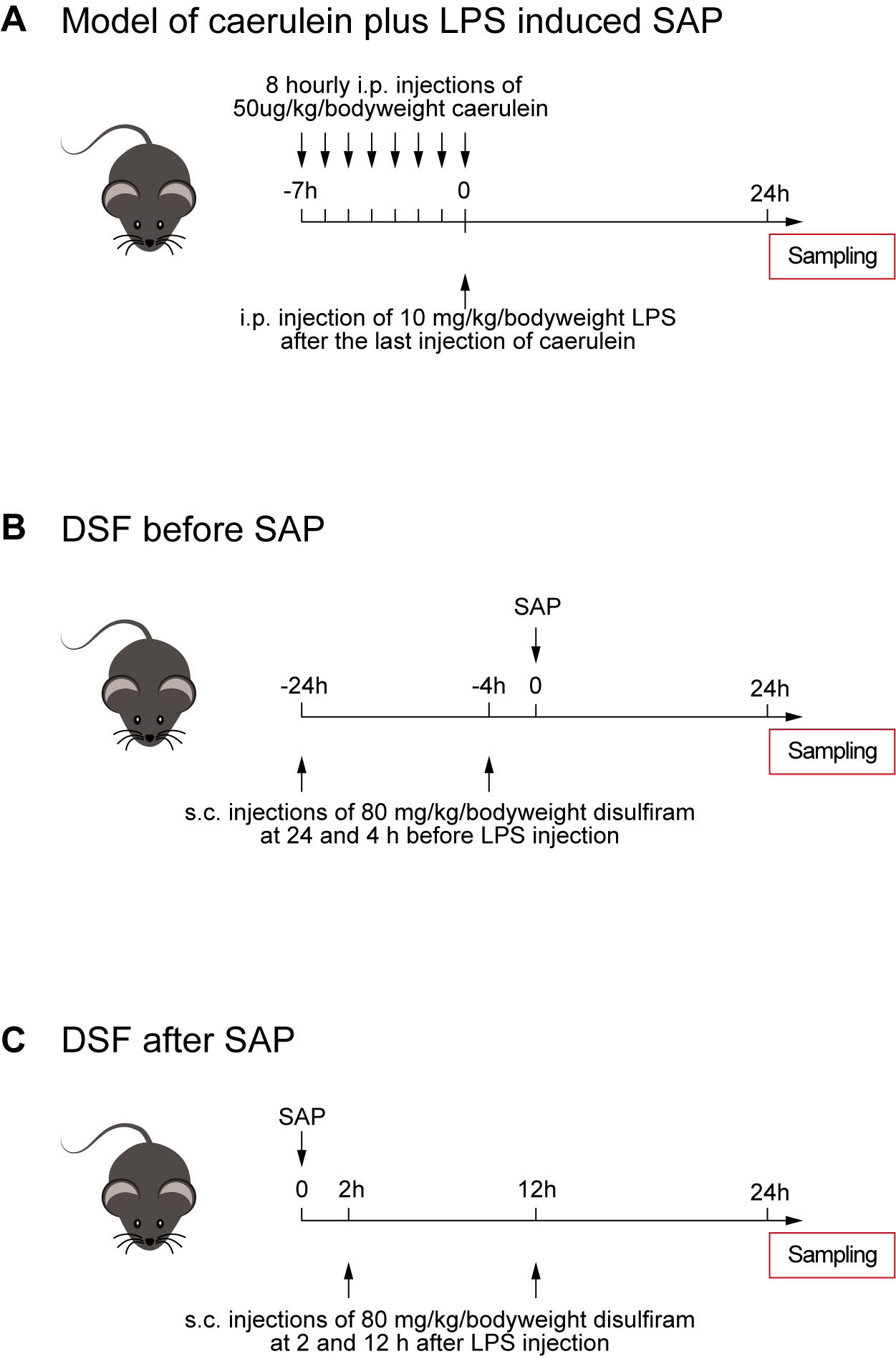

Supplement: Supplementary file 1 — FIGURE S1 Induction of experimental severe AP and treatments. (A) A schematic illustration of the severe acute pancreatitis model induced by caerulein plus LPS, (B) disulfiram was administrated before SAP, (C) disulfiram was administrated after SAP. [file JCMM-29-e70707-s001.tif]
