# Supplementary material for: Disulfiram Protects Against Multiorgan Injuries and Cell Pyroptosis via Inhibiting GSDMD in Severe Acute Pancreatitis Mice
Source: J Cell Mol Med. 2025 Aug 13;29(15):e70707. doi: 10.1111/jcmm.70707 (PMC12344577; doi:10.1111/jcmm.70707)
Supplement: Supplementary file 2 — Data S1. Supporting Information. [file JCMM-29-e70707-s002.docx]

**Supplementary materials and methods**

**Western Blot Analysis**

In brief, a total of 20 μg proteins were separated by 10% SDS-polyacrylamide gel electrophoresis and subsequently transferred to nitrocellulose membranes.

Primary antibodies used were rabbit anti-p-NF-κB p65 (3033, CST, 1:1000), rabbit anti-NF-κB p65 (8242, CST, 1:1000), rabbit GSDMD (ab209845, Abcam, 1:1000) and rabbit anti-GAPDH (5174, CST, 1:1000). The second antibodies were peroxidase-conjugated goat anti-rabbit and anti-mouse IgG (1:3000) (Elabsience, Wuhan, China). To visualize the proteomic bands, an enhanced western luminescent detection kit (4A Biotech, Beijing, China) was employed. The densitometry results were quantitatively analyzed by using the Image J software, with GAPDH bands being normalization/internal controls as appropriate.

**Real-time PCR Analysis**

Total RNA was extracted by Total RNA Extraction Reagent (Takara, 9109) according to the manufacturer’s instructions. cDNA was synthesized by Reverse Transcription Kit (Vazyme, R323-01). qPCR was performed using ChamQ Universal SYBR qPCR Master Mix (Vazyme, Q711-02). The amount of each cDNA relative to the GAPDH endogenous control was determined using the 2^-ΔΔCt^ method. All primers used are illustrated in **Table 1**.

**SYTOX Green nucleic acid staining**

SYTOX (MX4228, Maokangbio, Shanghai, China) was diluted in PBS to a final concentration of 1μM. Frozen sections were incubated in working solution for 1 hour and washed in 1×PBST for 10 minutes. Then the cryosections were stained with DAPI for 10 minutes at room temperature.

**Histological examination and biochemical assays**

Tissues were fixed with 4% paraformaldehyde overnight and then embedded in paraffin. H&E-stained sections were obtained and analyzed by pathologists in a double-blind manner.

Serum levels of AMY, ALT, TBIL, and BUN were determined by using the AMY, ALT, TBIL and BUN assay kit (Rayto, Shenzhen, China).

Serum levels of IL-1β, IL-6, and TNF-α were determined by using the IL-1β, IL-6, and TNF-α Elisa assay kit (NEOBIOSCIENCE, China).

**LDH assays**

Tissues were freshly isolated and homogenized. Measurement of LDH activity was performed according to the supplier’s instructions (LDH Assay Kit, BC0685, Solarbio, Beijing, China).

**Statistical Analysis**

The data were examined utilizing either the two-tailed Student' t test or the one-way ANOVA, followed by post-hoc t tests. Unless specified differently, all data is presented as the mean ± standard deviation. A statistical significance threshold of *p* < 0.05 (*) was adopted for all tests. GraphPad Prism Version 9.0.0 was utilized to conduct the statistical analysis.
